# Integrating Flow Cytometry in the Diagnostic Work of HIV‐Associated Hodgkin's Lymphomas

**DOI:** 10.1111/jcmm.71143

**Published:** 2026-05-05

**Authors:** Maria Santa, Diana Cenariu, Adrian‐Bogdan Tigu, Marc Damian, Patricia Rantshabeng, Tendani Gaolathe, Andrew K. Ndlovu, Khalid Abdelrahman, Madalina Nistor, David Kegyes, Diana Gulei, Cristian Jinca, Anamaria Bancos, Ioana Rus, Horia Bumbea, Victor Tomacinschii, Sanda Buruiana, Rand Bilal, Cristina Selicean, Mihnea Zdrenghea, Bogdan Fetica, Cristina Stefan, Jonathan Fromm, Ciprian Tomuleasa

**Affiliations:** ^1^ Department of Personalised Medicine and Rare Diseases, Medfuture Institute for Biomedical Research Iuliu Hațieganu University of Medicine and Pharmacy Cluj‐Napoca Romania; ^2^ Department of Haematology Iuliu Hațieganu University of Medicine and Pharmacy Cluj‐Napoca Romania; ^3^ Department of Haematology Ion Chiricuta Oncology Institute Cluj‐Napoca Romania; ^4^ Department of Pathology, Faculty of Medicine University of Botswana Gaborone Botswana; ^5^ Botswana‐Harvard Health Partnership Gaborone Botswana; ^6^ Department of Oncology Bistrita Emergency Hospital Bistrita Romania; ^7^ Department of Haematology University Emergency Hospital Bucharest Romania; ^8^ Department of Haematology Carol Davila University of Medicine and Pharmacy Bucharest Romania; ^9^ Department of Haematology Nicolae Testemitanu State University of Medicine and Pharmacy Chisinau Moldova; ^10^ Department of Haematology Public Medical Sanitary Institution (PMSI) Institute of Oncology Chisinau Moldova; ^11^ Department of Anatomy and Pathobiology Universidad Autónoma de Madrid Madrid Spain; ^12^ Institute for Global Health Equity Research University of Global Health Equity Kigali Rwanda; ^13^ Global Health Institute SingHealth‐Duke NUS Singapore Singapore; ^14^ Department of Laboratory Medicine and Pathology University of Washington Seattle Washington USA

**Keywords:** diagnostic work‐up, flow cytometry, HIV infection, Hodgkin's lymphoma

## Abstract

Hodgkin lymphoma (HL) is prevalent worldwide and typically presents symptoms like sudden pain, swelling, and weight loss. Classical HL (cHL) is largely treatable with modern risk‐adapted and response‐based therapy. cHL represents one of the most common malignancies occurring during the course of evolution in patients living with HIV. The present manuscript aimed to present the diagnostic work‐up with an emphasis on flow cytometry in patients with HIV‐associated lymphoma, of important clinical benefit both in the HIV‐endemic setting as well as in state‐of‐the‐art pathology departments. Three clinical cases of HIV patients, including HIV‐associated cHL, are presented in the chapter dedicated to HIV‐induced lymphomas diagnosed by FC, as Supporting Information, to support the particularities of the abundance of clonal B‐cell populations among different types of HIV‐related lymphomas. This adds to the proof‐of‐concept that the specific antibody panel design may help practitioners in discriminating among similar subtypes of HIV‐associated lymphomas. Recent data emphasise the importance of FC detection of cHL rapidly and effectively, adding diagnostic value to these small biopsies. FC is an important tool for clinical decision‐making in the management of HL patients, providing a noninvasive and accurate biomarker evaluation. For these considerations, we can conclude that FC is a very useful research tool, and the clinical cases presented in this paper indicate their importance in the rapid diagnosis of cHL as well.

## 1 Pathogenesis of Classical Hodgkin Lymphoma

### 1.1 Background on the Pathology of Hodgkin's Lymphoma

Classical HL (cHL) is largely treatable with modern risk‐adapted and response‐based therapy; however, the occurrence of relapsed or refractory disease, along with delayed treatment‐related toxicities, continues to pose considerable clinical difficulties [1, 2, 3, 4, 5, 6, 7, 8, 9, 10, 11, 12, 13, 14]. Differential diagnosis is challenging due to overlapping with other aggressive lymphomas, particularly diffuse large B‐cell lymphoma (DLBCL) and so‐called “grey zone” lymphomas; careful integration of morphology, immunophenotype, and molecular features is required [1, 2].

An enhanced comprehension of cHL biology has created a chance within WHO‐HEM5 to provide more detailed guidelines that differentiate cHL from its various diagnostic categories and similar conditions. Additionally, there is a growing acknowledgement of the overlaps between EBV‐positive cHL and DLBCL, though further data is required for precise classification, particularly regarding immune senescence. The comprehensive reevaluation that has resulted in the new WHO‐HEM5 guidelines for cHL carries wider implications for both clinical practice and epidemiological considerations moving forward, as well as for the ongoing refinement of future classifications, [3] typically positive for CD30 and variably for CD15, with weak PAX5 expression and negative for CD45 and B‐cell markers such as CD20 in most cases [4].

### 1.2 The Tumour Microenvironment (TME) of cHL

**FIGURE 1:**
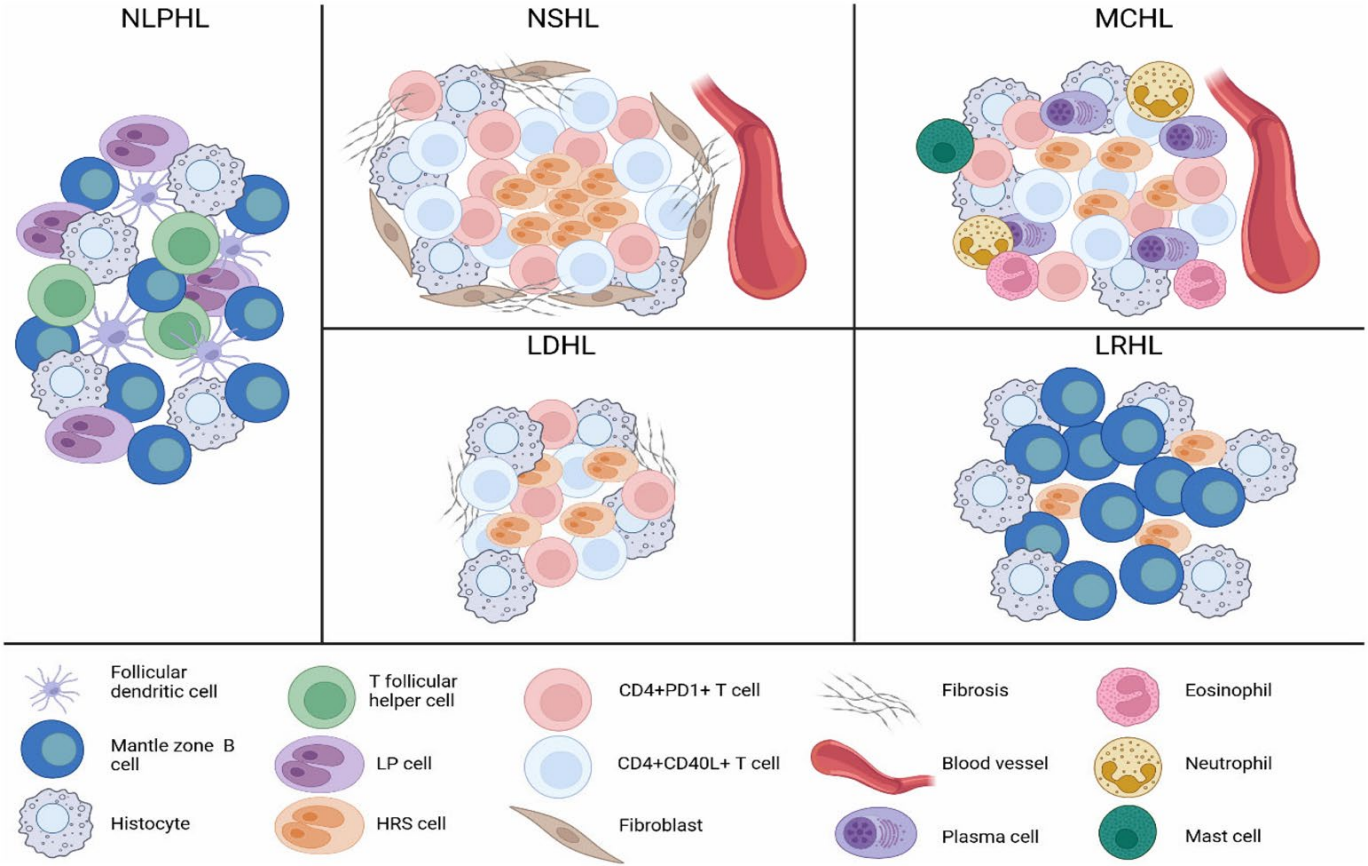
The cellular characteristics of Hodgkin Lymphoma. According to Connor et al.,17 HL classification includes classic HL (cHL) and nodular lymphocyte‐predominant HL (NLP‐cHL). cHL is further divided into four subtypes, with different cellular distribution: Nodular sclerosis HL (NS‐cHL), characterised by a TME with fibroblast‐like cells and fibrosis; mixed cellularity HL (MC‐cHL), with a TME characterised by a polymorphous reactive infiltrate of B and T cells, neutrophils, histocytes, mast cells and plasma cells; lymphocyte‐depleted HL (LD‐cHL), with the TME rich in histocytes and irregular fibrosis; and lymphocyte‐rich HL (LR‐cHL), with a variable TME but with histocytes and lymphocytes. On the other hand, NLP‐cHL is similar to the LR‐cHL with an increased presence of follicular dendritic cells. Figure 1 was created in BioRender.

In cHL, the tumour cells are referred to as Hodgkin and Reed‐Sternberg (HRS) cells and lymphocyte predominant (LP) cells in Nodular Lymphocyte Predominant Hodgkin Lymphoma (NLP‐cHL) (Figure 1). HRS cells actively manage their complex microenvironment through the secretion of cytokines and chemokines, attracting various immune cell subsets—such as T cells, eosinophils, macrophages, plasma cells, and fibroblasts—to the affected tissues. This recruitment supports their survival and proliferation while fostering an immunosuppressive environment [5] is notable for a proliferation of fibroblasts and myofibroblasts, which contribute to the formation of the fibrous bands. These fibroblasts exhibit a distinct phenotype, including upregulation of TIMP3, which likely contributes to the accumulation of collagen and the sclerotic bands seen in NS‐cHL [6].

HRS cells, which arise from pre‐apoptotic germinal centre (GC) B cells, have lost most of their B‐cell‐specific gene expression and surface markers, often displaying atypical markers for B cells such as CD30 and CD15, while still showing weak positivity for PAX5 [7, 8, 9]. Morphologically, HRS cells are large, possessing abundant cytoplasm and distinctive bi‐ or multinucleated nuclei, frequently described as having an “owl‐eye” appearance.

HRS cells activate several signalling pathways, particularly NF‐κB and JAK/STAT, which are pivotal for cell survival and proliferation. Genetic alterations in these pathways, including inactivating mutations in negative regulators like TNFAIP3, are common. Immune evasion is a notable feature, with frequent amplifications at chromosome 9p24.1, resulting in the overexpression of PD‐L1/PD‐L2, which allows HRS cells to evade immune detection [5, 10].

On the other hand, LP cells are known as “popcorn cells” due to their distinctive multi‐lobated or folded nucleus [11] and are the hallmark of Nodular lymphocyte predominant HL (NLP‐cHL). The LP cells are characterised by the presence of B‐cell markers, including CD20+, CD79a, Pax5, Oct‐2, Bob‐1, and J chain. In contrast, cHL markers CD30 and CD15 are negative. These cells seldom express EBV. NLP‐cHL typically exhibits a nodular growth pattern, with macro follicles that are enlarged due to follicular dendritic cells (FDCs). These networks can be easily recognised through FDC markers such as CD21 and CD23 [12]. Surrounding the malignant cells are T helper cells that express CD3, CD4, and CD57. Additionally, other markers commonly found on LP cells include BCL6 and EMA [12].

## 2 Pathogenesis of HIV‐Associated HL

**FIGURE 2:**
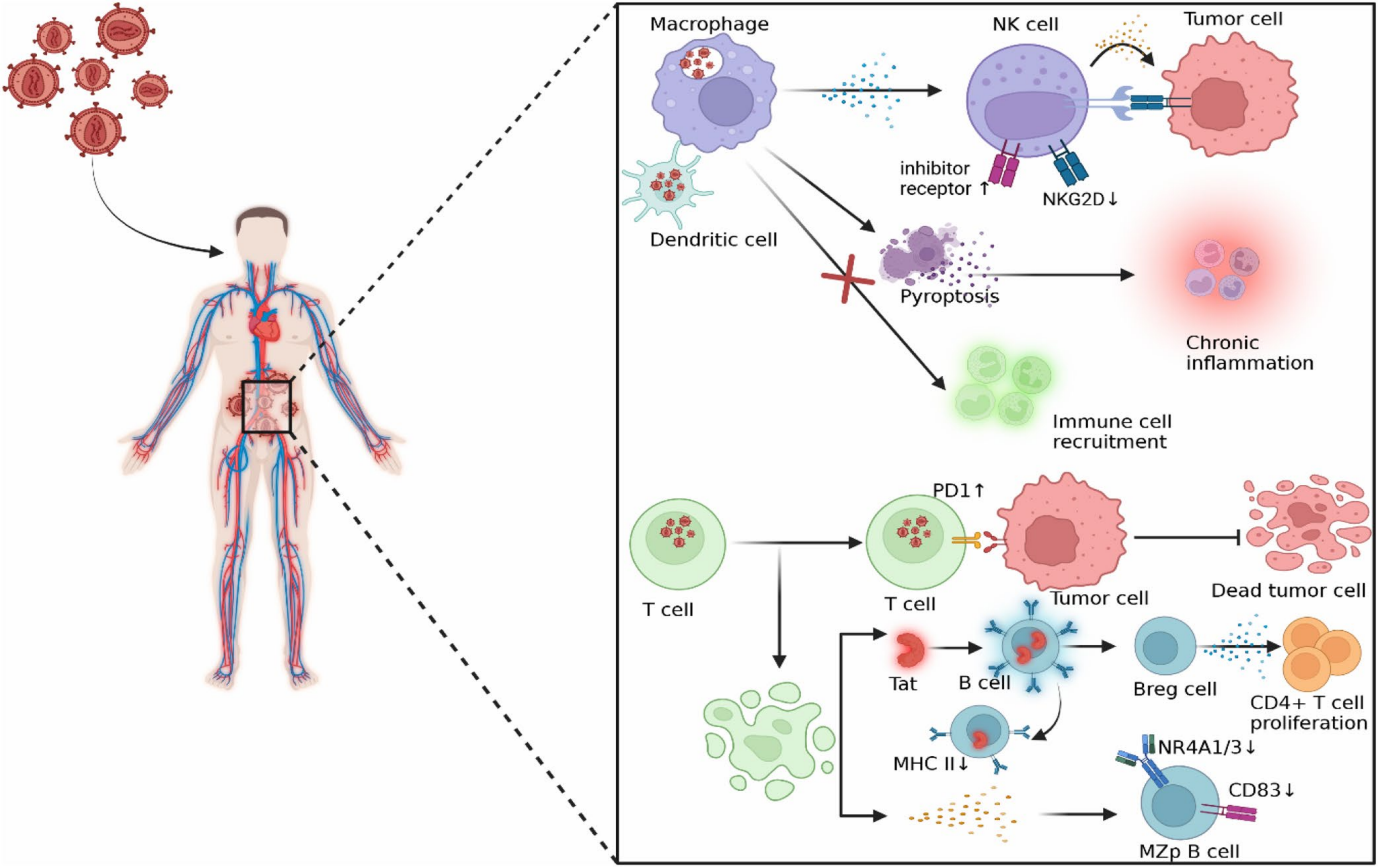
Immune Characteristics of HIV‐Associated Lymphoma. HIV infection could trigger lymphomagenesis through several mechanisms. (A) by inducing T cell depletion by PD‐1 overexpression and reducing immune surveillance, and by impairing B‐cell function through Tat‐ induced NF‐kB pathway inhibition and increasing BAFF. (B) Through chronic inflammation and immune dysfunction induced by HIV‐infected macrophages that impair BST2 and initiate pyroptosis, and by suppressing NK cell activity through NKG2D downregulation and upregulating inhibitory receptors. The figure is an original figure created in BioRender.

HIV plays a significant role in the development of HL by causing immune dysregulation, persistent activation of B cells, and reduced immune surveillance, while also promoting lymphomagenesis linked to Epstein‐Barr virus (EBV) (Figure 2) [13, 14, 15].

HIV‐associated Hodgkin lymphoma (HIV‐HL) represents a significant malignancy in people living with HIV (PLWH), with a markedly elevated incidence compared to the general population. People with HIV are 5 to 14 times more likely to be diagnosed with classical Hodgkin lymphoma (cHL) than HIV‐negative individuals, and cHL accounts for approximately 4% of all cancers diagnosed in the HIV population. According to the National Comprehensive Cancer Network database, recent global estimates indicate that PLWH have an 11.51‐fold increased risk of developing Hodgkin lymphoma compared to the general population [16]. In the US, the standardised incidence ratio (SIR) for Hodgkin lymphoma in PLWH was 6.29 from 2015 to 2019, representing a 17% reduction in relative risk compared to the 2010–2014 period PMID: 40504560. The incidence of HIV‐HL has shown a paradoxical trend in the era of combination antiretroviral therapy (cART). Unlike aggressive non‐Hodgkin lymphomas, which decreased substantially after the introduction of cART, the incidence of Hodgkin lymphoma increased from 30 per 100,000 person‐years in the pre‐cART era (1980–1995) to 49 per 100,000 person‐years in the early cART era (1996–2002) [17]. Subsequently, incidence rates have remained relatively stable or shown modest declines [18]. Globally, 6.07% of new Hodgkin lymphoma cases in 2019 were attributable to HIV infection, representing a 2.84‐fold increase from 1990 [16]. Regional variations are substantial, with Eastern and Southern Africa showing the highest burden, where 41.13% of incident Hodgkin lymphoma cases are attributed to HIV infection [16].

The relationship between HIV‐mediated immunosuppression and Hodgkin lymphoma risk is complex and differs from other HIV‐associated malignancies. Studies have shown that Hodgkin lymphoma incidence peaks at moderate immunosuppression (CD4 counts of 225–249 cells/μL) at 73 per 100,000 person‐years, and paradoxically decreases to 27 per 100,000 person‐years in patients with profound immunosuppression (CD4 25 cells/μL) [17]. This unique pattern may reflect complex interactions within the tumour microenvironment between Epstein–Barr virus (EBV), non‐neoplastic T‐lymphocytes, and neoplastic Reed‐Sternberg cells [17]. Notably, patients may experience progressive loss of CD4 cells during sustained HIV suppression on cART, with cases losing an average of 98 CD4 cells in the year before diagnosis, suggesting that declining CD4 counts despite viral suppression may harbour occult Hodgkin lymphoma [19].

The pathogenesis of HIV‐associated Hodgkin lymphoma is fundamentally linked to EBV infection, which occurs in 80%–100% of HIV‐HL cases compared to only 20%–40% of Hodgkin lymphoma in the general population, according to the National Comprehensive Cancer Network database [17, 20]. This near‐universal EBV association represents one of the most distinctive features of HIV‐HL and plays a central role in lymphomagenesis. EBV demonstrates a latency type II infection pattern in HIV‐HL, characterised by expression of latent membrane protein‐1 (LMP‐1) and LMP‐2A in Hodgkin and Reed‐Sternberg (HRS) cells [20]. LMP‐1 functions as a constitutively activated tumour necrosis factor receptor‐like molecule that mimics CD40 activation, usurping physiologically relevant signalling pathways involved in cell activation, growth, and survival [20]. Through LMP‐1 expression, EBV activates multiple oncogenic pathways, including NF‐κB, JAK–STAT, and c‐MYC, promoting proliferation and survival of malignant cells while simultaneously facilitating immune evasion [10, 21, 22, 23].

The cooperation between HIV and EBV creates a unique pathogenic environment. HIV‐induced immunosuppression impairs immune surveillance and allows EBV to proliferate in infected B cells [22, 24]. HIV contributes to lymphomagenesis through T‐cell depletion and dysregulation, B‐cell dysregulation, cytokine dysregulation, and creation of a chronic pro‐inflammatory state [22, 24]. The tumour microenvironment in HIV‐HL is characterised by compromised adaptive immune reactivity, with higher densities of CD8+ T cells coexpressing inhibitory receptors (PD‐1 and TIGIT), altered macrophage subsets, and dysregulated T‐cell receptor signalling pathways [25].

Despite improvements in combination antiretroviral therapy, co‐infection with HIV accelerates disease progression, promotes M2 macrophage dominance, and results in T‐cell depletion, while the interactions with other viral infections remain unclear. EBV induces clonality in HRS cells, enhancing their survival by inhibiting apoptosis and increasing PDL1 expression. This process is driven by the viral protein latent membrane protein 1 (LMP1), [26] which increases the expression of CXCR2, a receptor for HIV p17 [27] and enhances CD40 activation and promotes proliferation [28].

### 2.1 Latent Membrane Protein 1 (LMP1) Expression in HRS Cells

The expression of LMP‐1 leads to the upregulation of discoidin domain receptor 1 (DDR1) in GC B lymphocytes (precursors of HRS cells), improving their adhesion to collagen and reducing their commitment to programmed cell death [29] The interaction between tumour cells and their microenvironment contributes to lymphomagenesis by activating key intracellular signalling pathways, primarily the NF‐kB, JAK/STAT, and MAP/ERK pathways (Table 1).

**TABLE 1:** Dysregulated signalling pathways detected in cHL.

No.	Signalling pathway	Dysregulated pathway	Molecular Impact	References
1	NF‐kB Nuclear Factor kappa B in HRS cells BCL3 (Boron trichloride and B‐cell lymphoma 3 protein) a nuclear cofactor in the NF‐κB	Genetic lesions in REL, mutations in TNFAIP3, NFKBIA, and NFKBIE, and gains of IKBKB, CD40, and MAP3K14 HRS cells lack BCR signalling IgHV locus‐associated chromosomal translocations	Constitutive activation of NF‐kB promotes cell survival, proliferation, and resistance to apoptosis	[30, 31]
2	JAK/STAT (Janus kinase)/(signal transducer and activator of transcription)	Amplifications of JAK2 and mutations in SOCS1 Loss of negative regulators such as SOCS1, and ubiquitous mutations in JAK2, STAT6 Recurrent mutations in several genes, most notably GNA13, XPO1, ITPKB, STAT6 Copy number gains and 9p24 alterations JAK2 is localised near PDL1/2 involved in amplifications of the 9p24 region	Persistent activation of JAK/STAT, driving cell growth and survival Persistent activation of STAT3 and STAT6, supporting proliferation and immune evasion Constitutive activation of JAK/STAT cascade in almost 90% of cases Inferior outcome in conventionally treated patients	[32, 33]
3	PI3K/AKT/mTOR signalling (Phosphoinositide3‐kinase)/(Protein Kinase B)/ (mammalian Target of Rapamycin)	Aberrantly activated in approximately 45% of cHL GNA13 inhibits AKT phosphorylation and sustains proliferating germinal centre B cells	Apoptosis resistance and enhanced survival of HRS cells Inactivation of ITPKB mutations, in over 25% of cHL	[34, 35]
4	NOTCH1 Neurogenic locus notch homologue protein 1SPEN (SMARCA2/4‐binding protein) FRROM notch SIGNALLING	NOTCH abnormally active in HRS cells Alterations in SPEN (12.5%), NOTCH1/2 (2.5%, respectively) and FBXW7 (7.5%)	Negative regulator of the B‐cell program, loss of B‐cell phenotype and cellular reprogramming Mutated in up to 20% of the cHL	[36, 37]
5	The Tumour Necrosis Factor (TNF) family signalling CD30TNFRSF8 (Tumour Necrosis Factor Receptor Superfamily member8) and CD40TNFSF5 (Tumour Necrosis Factor Receptor Superfamily member5) signalling TNFRSF14 (Tumour Necrosis Factor Receptor Superfamily member 14) TNFAIP3 (tumour necrosis factor alpha‐induced protein 3) or A20	They are molecular markers of HRS cells, overexpressed on HRS cells Insure the survival and proliferation of HRS cells within the TME CD30 suppresses c‐Myc induces the CCR7 molecule, causing its Induction and up‐regulation Interconnection of CD30 and CD40 signalling network Somatic copy number loss of TNFRSF14 KM‐H2 cell line displays reduced TNFRSF14 by flow cytometry Immune evasion by disrupting normal T‐cell costimulatory signalling Genetic inactivation of the TNFAIP3 gene	CD30 is involved in Homing of lymphocytes to lymph nodes Apoptosis, and promote immune evasion Targeted therapies: Immunotherapies and checkpoint inhibitors Supports the aggressive nature of HL Deletions or point mutations inactivation of A20 constitutive NF‐κB activation in HRS cells	[30, 32, 38, 39, 40]
6	MAPK/ERK (Mitogen‐Activated Protein Kinase/Extracellular Signal‐Regulated Kinase)	TRAFs activate kinases of the MAPK pathway Stimulation of CD30, CD40, and RANK has been shown to induce phosphorylation of ERK, p38 and JNK kinases in cHL cell lines	Inhibition of MAPK activity leads to the cell cycle arrest and apoptosis of HRS cells Upregulation of CD30, CD 40 in HRS cells by activation of MAPK/ERK pathway, results in JunB (Jun proto‐oncogene) expression	[41, 42, 43, 44]
7	Programmed cell death protein 1/programmed death‐ligand 1 (PD‐1/PD‐L1) axis PD‐1/CD279 Two ligands PD‐L1 (CD274) or B7‐H, and PDL2 (CD273) or B7‐DC	Is upregulated in HRS cells, Alterations of chromosome 9p24.1 Overexpression of genes encoding PD‐L1 (CD274) and PD‐L2 (PDCD1LG2), enhanced by JAK2 signalling	Induces CD8^+^ cytotoxic T‐cell exhaustion Results in an immunosuppressive niche and escape of immune surveillance	[5, 10, 45, 46, 47]
8	Activator protein‐1 (AP‐1)	Constitutively activated in HRS cells Regulates genes such as PD‐L1 and Galectin‐1 Cross‐Talk between AP‐1 and Cell Surface Proteins, CD15 and CD30 Involvement of AP‐1 TFs in the Pathogenesis of CD30+ Peripheral T‐Cell Lymphomas (PTCLs)	High levels of retroviral onco‐proteins c‐Jun/JunB and c‐FOS Promotes tumour cell proliferation, supports cell survival, and contributes to the immunosuppressive tumour microenvironment	[5, 45, 48, 49, 50, 51]
9	Beta‐2 microglobulin (β2M)	It impairs major histocompatibility complex (MHC) class I molecules expression and the ability of CD8+ T cells to recognise and eliminate HRS cells Elevated serum β2M levels in cHL patients	Facilitates immune evasion of HRS cells Higher tumour burden, advanced stage, and inferior outcomes	[52, 53, 54, 55]

### 2.2 Epstein–Barr Virus‘s Impact on the TME

Recent multi‐omics investigations into HIV‐associated Hodgkin lymphoma revealed a compromised TME and concluded that viral infections (EBV and HIV/EBV) do not significantly alter the densities of neoplastic CD30‐high cells in cHL tissues.

EBV has the capability to infect a range of cell types, enabling them to activate growth programs and sustain proliferation, a phenomenon that is usually controlled by cytotoxic T lymphocytes (CTLs) [25]. When the CTL response is weakened, these cells can progress to post‐ transplant lymphoproliferative disorder (PTLD) or lymphomas associated with HIV [21, 56]. Distinctions between EBV‐positive and EBV‐negative cHL are significant, as EBV‐ positive cHL is generally associated with mixed cellular HL (MC‐cHL) and lymphocyte‐depleted HL (LD‐cHL), characterised by a dense inflammatory background filled with numerous CD68+ and CD163+ macrophages, cytotoxic T lymphocytes, and frequent necrotic areas [57] In contrast to non‐HIV cHL, the TME in HIV‐associated cHL is enriched with CD68+ and CD163+ spindle M2 macrophages, PD‐L1 and Galectin‐1‐expressing cells, and significantly lower levels of CD4+ T cells, CD56+ NK cells, CD57+ cells, CD123+ dendritic cells, and B cells, creating a potent immunosuppressive environment that protects HRS cells from immune attacks [7].

EBV plays a critical role in promoting the survival of cancer cells, modifying immune responses, and enabling mechanisms of immune evasion. The standardised diagnostics published by the WHO 2024 [3] emphasised the adequate characterisation of the immune environment, as well as EBV latency patterns, which can improve prognosis and inform patient‐tailored therapies, and enhance understanding of the interplay between genetic alterations, viral pathogenesis, and immune dysregulation in cHL [10, 28].

## 3 FC For HIV‐Associated HL

The advantages of using FC for lymphoma diagnosis and monitoring include rapid, multiparametric immunophenotyping of lymphoid cells, which enables sensitive and specific identification of abnormal populations, even in small or low‐cellularity samples such as fine needle aspirates or core biopsies [58, 59]. FC can simultaneously analyse multiple antigens on individual cells, facilitating precise classification of lymphoma subtypes and detection of clonality, particularly in B‐cell and T‐cell neoplasms [60, 61]. This technique is highly quantitative, allowing for objective assessment of antigen expression, cell size, granularity, and DNA content, which supports disease classification, staging, and prognostic marker evaluation [62, 63]. FC is also the method of choice for MRD detection in certain lymphoma entities, providing high sensitivity for submicroscopic disease and independent prognostic information [64]. Additionally, it enables rapid triage for ancillary studies, such as molecular or cytogenetic testing, and can be used to monitor treatment response and guide personalised therapy, including immunotherapies.

Compared to immunohistochemistry, FC offers superior multiparametric analysis, faster turnaround, and the ability to evaluate both surface and intracellular antigens in single‐cell suspensions [65, 66]. Its utility extends to staging, central nervous system involvement assessment, and detection of therapeutic targets or multidrug resistance markers. Overall, FC could serve as a supportive tool in the modern diagnostic and monitoring workflow for lymphomas, especially as clinical practice shifts toward less invasive sampling.

FC for lymphomas should be of global interest in oncology because it is a high‐precision tool for rapid, multiparametric immunophenotyping, enabling accurate diagnosis, classification, and prognostication of lymphoid neoplasms, especially in settings where tissue samples are limited or minimally invasive procedures are preferred [59, 67]. The technique allows for the simultaneous analysis of multiple antigens on individual cells, facilitating the identification of clonal B‐ and T‐cell populations, distinguishing between indolent and aggressive subtypes, and supporting the World Health Organization classification of haematolymphoid neoplasms [62]. The American Society for Clinical Pathology and the College of American Pathologists recommend FC as a core component of the laboratory workup for lymphoma, particularly for small‐volume specimens such as fine needle aspirates and core biopsies, which are increasingly used worldwide due to trends toward less invasive diagnostics [67]. FC also provides critical information for triaging ancillary studies, monitoring minimal residual disease, and evaluating prognostic markers such as DNA ploidy and proliferation fraction, which can inform treatment decisions and risk stratification [68]. Given its speed, sensitivity, and ability to guide precision medicine approaches, including immunotherapy selection and monitoring, FC is essential for modern lymphoma management across diverse healthcare systems and resource settings. Its global relevance is underscored by its utility in both high‐resource and low‐resource environments, where rapid, accurate, and minimally invasive diagnostics are increasingly necessary for optimal patient care.

FC serves as a valuable supplementary tool in diagnosing Hodgkin lymphoma, particularly cHL. FC is considered a valuable medical tool, complementary to histopathology and immunohistochemistry. However, there are studies by cytometry experts that have brought the technique to a diagnostic value, for Hodgkin's Lymphoma [58, 69]. Histopathology and immunohistochemistry remain the gold standard, largely because HRS cells are scarce (less than 1% in lymph nodes), frail, and located within a complex inflammatory microenvironment, composed of eosinophils, plasma cells, histiocytes and reactive lymphocytes [70, 71, 72].

### 3.1 How I Integrate FC in the Management of HIV‐Related—Classic Hodgkin Lymphoma

Multiparametric FC can identify HRS cells and their characteristic immunophenotype (CD30+, CD15+, CD20 and weak/variable CD45 and often CD123+), as well as the detection of associated T‐cell rosettes, both in tissue and cytological specimens. Fromm et al. have indicated that T cells bind to HRS cells, resulting in a “rosette” that (when immunophenotyped by FC) shows a composite immunophenotype of the T cells and Hodgkin cells (CD5+, CD15+, CD20‐, CD30+, CD40+, CD45+ (bright), CD64‐, CD71+, CD95+). While unlabeled “blocking” antibodies that disrupt the interaction of adhesion molecules on the surface of HRS cells (CD54 and CD58) and T cells (LFA‐1 and CD2), the use of blocking antibodies in clinical practice is unnecessary. Additionally, detection of the presence of T cell–HRS cell rosettes is diagnostically useful. FC images came from Fromm's patient database (Department of Laboratory Medicine, University of Washington, Seattle, USA), representing original FC plots [73].

HRS cells are characterised by being CD64 negative and expressing CD30, CD40, and CD95, along with an increased Side Light Scatter (SSC‐H) when compared to normal lymphocytes. These cells consistently show expressions of CD30, CD40, CD71, and CD95, while CD15 is present in most cases. The expression levels of CD40 and CD95 on HRS cells are frequently higher than those found in other cellular populations within the lymph node.

cHL in HIV+ patients can present with advanced‐stage disease with bone marrow or liver involvement. EBV‐positive cHL in HIV+ patients can be immunophenotyped (IP) by FC assay evaluation of CD30+, CD15+, PAX‐5+, CD3‐, CD20‐, CD45‐ [52] (Supporting Information 1).

**FIGURE 3:**
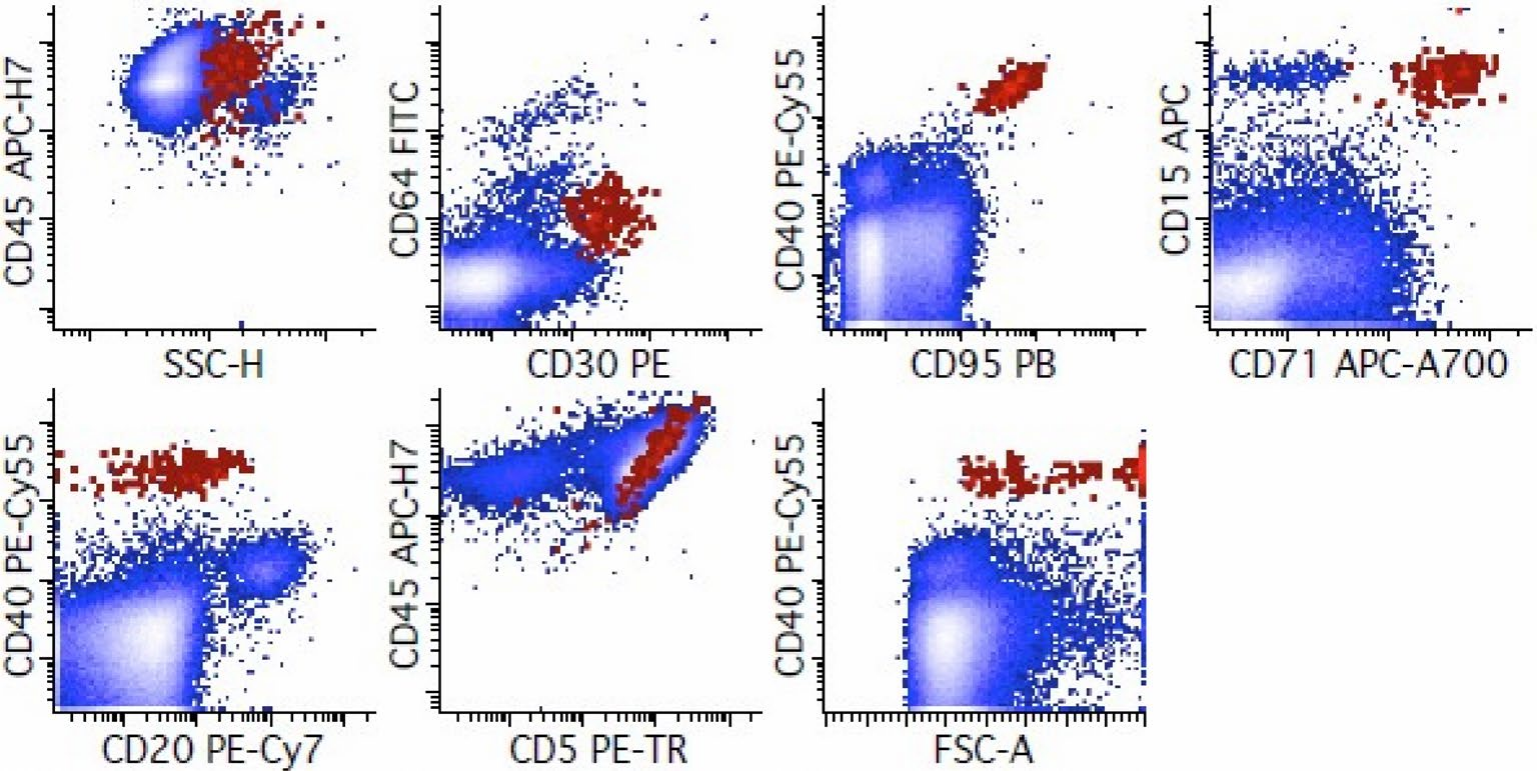
IP by Flow Cytometry for diagnosis of Classic Hodgkin lymphoma. The method used to identify HRS cells was cell suspensions from lymph node biopsies using a 2‐tube, FC assay to diagnose cHL (88% sensitivity and 100% specificity) when analysing an adequate specimen. The detailed method of FC tube preparation can be consulted as previously shown [73] (according to supplemental material 1).

The neoplastic population of HRS cells exhibit intermediate positivity for CD15, variable and intermediate‐to‐strong expression of CD20, as well as intermediate levels of CD30, CD40, CD71, and CD95, while remaining negative for CD64, as shown in Figure 3, from the flow cytometry personal database of Jonathan Fromm, University of Washington, Seattle, USA. The observed diagonal relationship between CD45 and CD5 is attributed to T‐cell–HRS cells. Neoplastic cells are generally CD19− and CD20−, although they may still express these B cell antigens at rates of 29% and 35%, respectively. FC reveals a higher expression of CD45 on HRS cells compared to immunohistochemistry. For a better understanding of the importance of FC evaluation, some laboratories plan to start using this instrument for predicting HL outcome as a prognosis tool. The lymph node populations [53], especially T‐cell–HRS cell rosettes involvement, are identified by FC and associated with a less/more aggressive disease outcome: More rosettes present, less severity and cHL remission [58, 74].

FC offers quick, sensitive, and precise immunophenotypic data, particularly beneficial for small biopsies or cytology when tissue samples and excisional biopsies are limited. Recent studies indicate that FC can achieve sensitivity levels as high as 95% and specificity up to 98% for classical Hodgkin lymphoma [6, 45]. In these scenarios, faster diagnosis is facilitated, and the necessity for more invasive procedures can be excluded. FC can identify HRS cells by their large size, high side scatter, and unique immunophenotype, but detection is challenging due to their scarcity and fragility. The background in cHL is rich in reactive T cells, especially CD4+ T cells, often with an increased CD4:CD8 ratio and overexpression of CD7 on CD4+ T cells. Some studies have also shown increased CD71 expression on CD4+ T cells in cHL [32, 48, 65, 75].

### 3.2 How I Integrate FC in the Management of HIV‐Related—DLBCL

**FIGURE 4:**
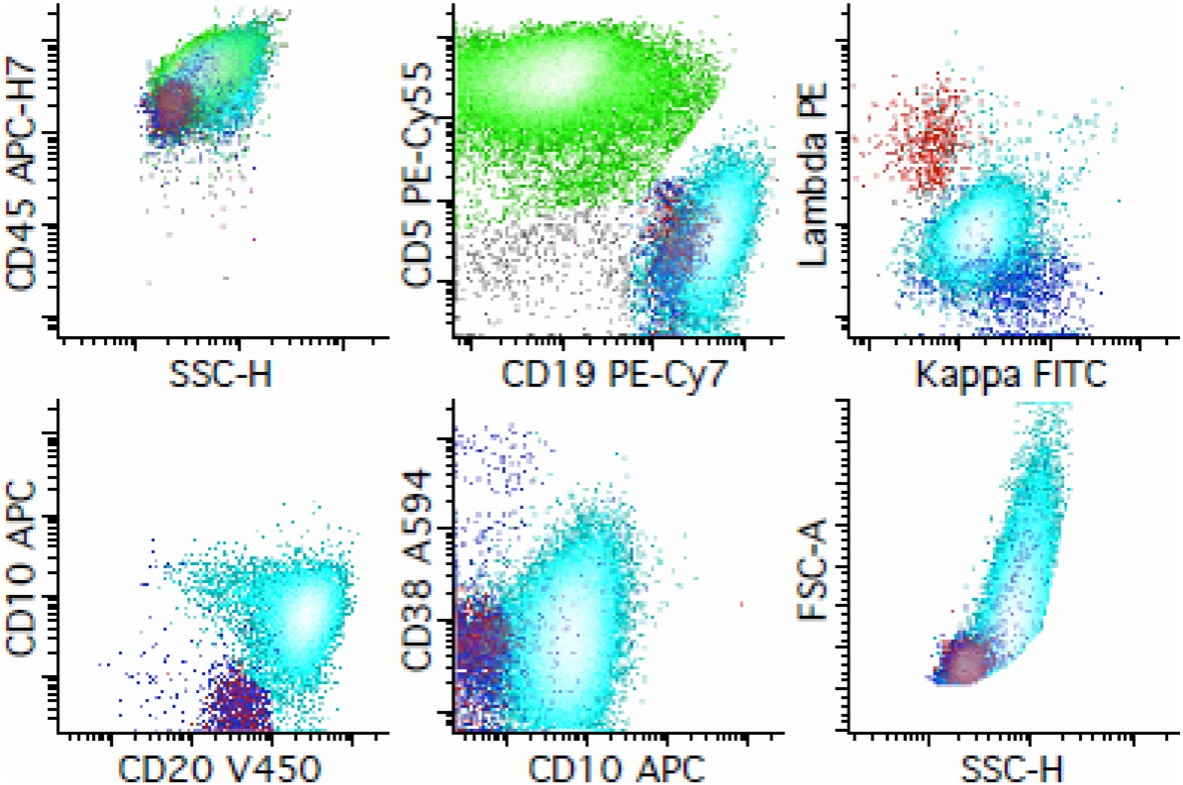
IP by Flow Cytometry for diagnosis of DLBCL.

Non‐Hodgkin B‐cell lymphomas show a dominant clonal B‐cell population with strong expression of B‐cell markers and distinct patterns. For DLBCL, the neoplastic cells are abundant and easily detected. In HIV+, often present with extranodal (CNS, GI tract, bone marrow) or disseminated disease; often high stage. The characteristic morphology of DLBCL is “centroblastic” or “immunoblastic”, presenting large B cells (twice the size of a normal lymphocyte) arranged in a diffuse pattern. IHC of centroblastic DLBCL (as shown in Figure 4, from the flow cytometry personal database of Jonathan Fromm, University of Washington, Seattle, USA) expresses pan B cell markers (CD19, CD20, cCD79a, PAX‐5), CD10+/‐, BCL6+/‐, CD138‐. IHC of immunoblastic (plasmacytoid) DLBCL: CD20+, CD10‐, BCL‐6‐, MUM1+, CD138+, CD38+. DLBCL may be of germinal centre or non‐germinal centre origin (germinal centre origin is most common), with ~30% EBV+ [52]. CD19+, CD20+, CD79a+, CD45+, variable CD10, BCL6+, usually negative for CD5 and CD23, and light chain restriction [49, 59] (Supporting Information 2).

### 3.3 How I Integrate FC in the Management of HIV‐Related—Burkitt's Lymphoma

**FIGURE 5:**
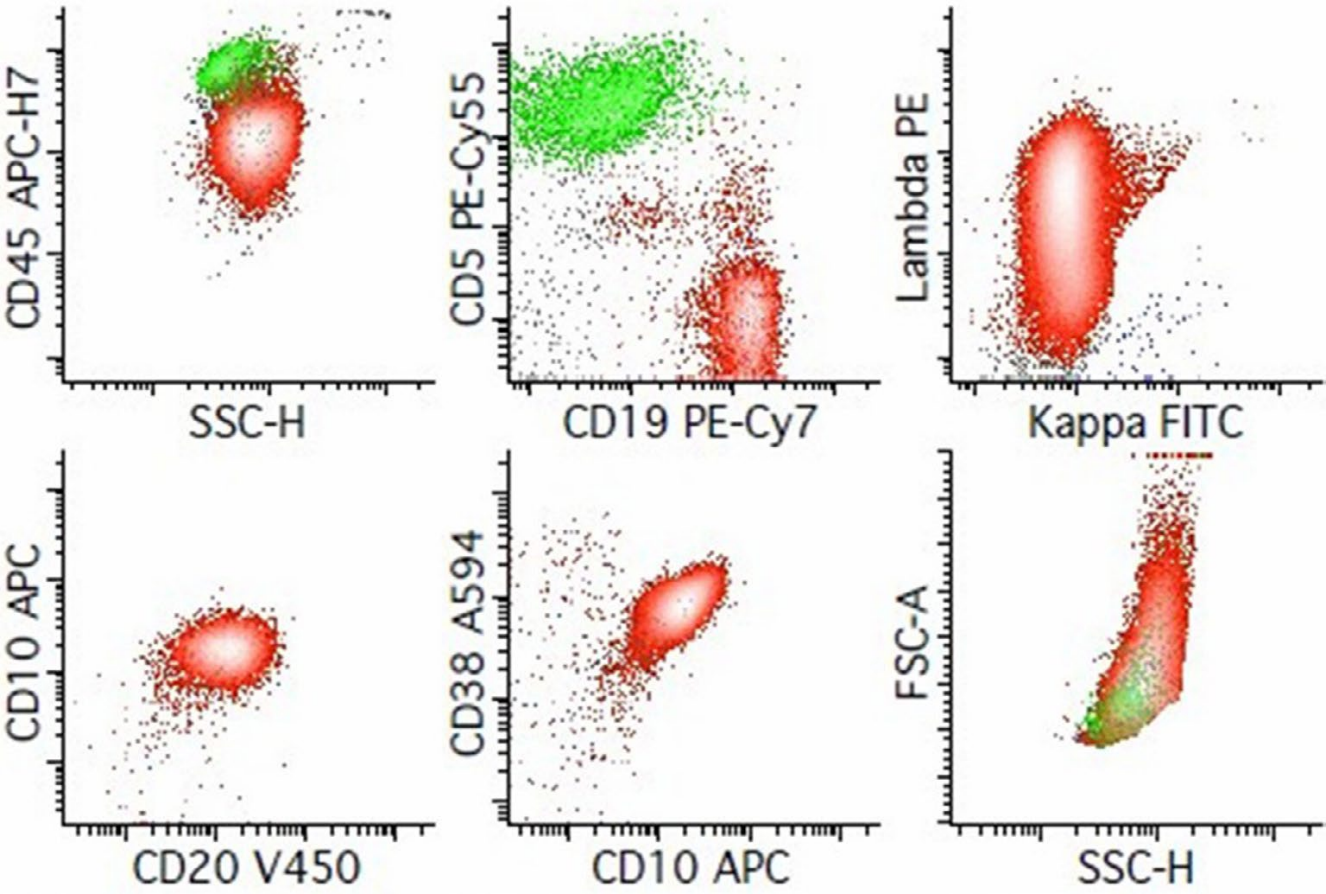
IP by Flow Cytometry for diagnosis of Burkitt lymphoma.

In Burkitt's lymphoma (BL), the following markers are expressed: CD19+, CD20+, CD10+, BCL6+, CD45+ (as shown in Figure 5, from the flow cytometry personal database of Jonathan Fromm, University of Washington, Seattle, USA), strong surface IgM with light chain restriction, CD38 bright, CD5–, CD23–, BCL2–, and nearly 100% Ki‐67. The background is not T‐cell rich [65]. High proliferative B cell lymphoma of intermediate‐sized cells showing a “starry sky” appearance at low power, 30%–50% EBV positive (Supporting Information 3) [65].

### 3.4 How I Integrate FC in the Management of HIV‐Related THRLBCL, NLPHL and PMBCL Lymphomas

1. T‐cell/histiocyte‐rich large B‐cell lymphoma—THRLBCL

cHL is defined by CD30+/CD15+ neoplastic cells with diminished B‐cell markers and a T‐cell‐rich background, while THRLBCL is defined by CD20+/CD45+ neoplastic B cells (CD15–/CD30–) in a background of abundant T cells and depleted B cells. These immunophenotypic and background differences are diagnostically useful in FC. FC can distinguish cHL from other lymphomas with similar characteristics, like nodular lymphocyte‐predominant Hodgkin lymphoma (NLPHL), T‐cell/histiocyte‐rich large B‐cell lymphoma (TCRLBCL) and Primary mediastinal large B‐cell lymphoma (PMLBCL), by analysing both neoplastic and basal cell type populations.

In THRLBCL, the neoplastic cells are large B cells that are CD20+, CD45+, BCL6+, CD75+, and negative for CD15 and CD30. These cells often lack surface immunoglobulin and may show overexpression of CD40, CD54, and variable expression of CD58 and PD‐L1/PD‐L2. The neoplastic B cells are also rare and scattered, but can be detected by FC with appropriate gating strategies. The background is characterised by a marked predominance of T cells, often with a high CD4:CD8 ratio, and a significant reduction in background B cells. Some cases show profound systemic B‐cell lymphopenia. The T‐cell compartment may also show increased CD57 and PD‐1 expression on CD4+ T cells [48, 76].

2 Nodular lymphocyte‐predominant Hodgkin lymphoma—NLPHL

NLPHL neoplastic cells (LP or “popcorn” cells) are CD20+, CD45+, BCL6+, CD79a+, and negative for CD15 and CD30. They retain a germinal centre B‐cell phenotype and often express surface immunoglobulin (IgD and/or IgM). The background is rich in small B cells and a characteristic population of CD4+CD8+ double‐positive, PD‐1 bright T cells, and increased CD57+ T cells, which are highly specific for NLPHL. The CD4:CD8 ratio is also increased, but the presence of these double‐positive and PD‐1 bright T cells is a distinguishing feature [32, 77].

3 Primary mediastinal large B‐cell lymphoma—PMBCL

PMBCL shows a neoplastic population with a clear B‐cell immunophenotype: Strong expression of CD19, CD20, CD79a, and CD45, with frequent expression of CD30, but usually negative for CD15. PMBCL cells often lack surface immunoglobulin light chains and may overexpress CD40. The background is less T‐cell rich than cHL, and the CD4:CD8 ratio is typically lower, with a relatively increased CD8+ T‐ cell population compared to cHL. PMBCL neoplastic cells are more readily detected by FC due to their robust B‐cell marker expression and higher abundance [78, 79].

cHL neoplastic cells are CD30+, CD15+ (often), weak/negative for B‐cell markers, and negative for CD45, with a T‐cell‐rich background (high CD4:CD8 ratio), while PMBCL neoplastic cells are CD19+, CD20+, CD45+, often CD30+, usually CD15–, and have a less T‐ cell‐rich background with a lower CD4:CD8 ratio. The available FC data can help disseminate an abnormal population of non‐Hodgkin B‐ or T‐cells from classical Hodgkin lymphoma, identified by the presence of a Hodgkin and Reed‐Sternberg (HRS) cell population.

### 3.5 FC Markers for HIV‐Associated Lymphoproliferative Disorders

Thus, the key differentiators are the lack of a dominant clonal B‐cell population and the characteristic immunophenotype of HRS cells in cHL, vs. the presence of a dominant clonal B‐cell population with strong B‐cell marker expression and light chain restriction in non‐Hodgkin B‐cell lymphomas. The background T‐cell population is also more prominent and skewed in cHL. Together with morphological and immunohistochemical information, FC is essential for an accurate diagnosis [48, 58, 76] cHL lacks a dominant clonal B‐cell population, whereas HIV‐related lymphomas show a clear, often abundant, clonal B‐cell population [80]. The presence of a dominant clonal B‐cell population with strong B‐cell marker expression and light chain restriction supports HIV‐related lymphoma, while the combination of rare CD30+/CD15+ HRS cells (often CD123+), weak/absent B‐cell markers, and a T‐cell‐rich background with increased CD4:CD8 ratio and CD71+ CD4+ T cells supports classic Hodgkin lymphoma [24].

In HIV patients who develop classic Hodgkin lymphoma, FC cell surface marker expression of Hodgkin and Reed‐Sternberg (HRS) cells is generally similar to that seen in HIV‐negative individuals: HRS cells remain CD30+, often CD15+, dim PAX5+, and negative or weak for B‐cell markers (CD20, CD19, CD79a) and CD45.

HIV‐associated cHL is characterised by a high level of depletion of CD4+ T cells, while the CD8+ T cells and CD163+ macrophages become relatively higher in the tumour microenvironment, resulting in an inverted or very low CD4:CD8 ratio. In contrast, the characteristic T‐cell‐rich background with an elevated CD4:CD8 ratio can be found in immunocompetent patients with cHL. Additionally, there is a higher frequency of EBV positivity in HRS cells in HIV‐related cases, with nearly universal expression of EBV latent membrane protein‐1 (LMP‐1) [7].

These changes in the microenvironment are the most characteristic findings which separate HIV related from non– HIV related cHL on FC. Patients with HIV or other immunosuppression, who have been treated with immune‐modifying agents, may present atypical expression of cell‐surface antigens by FC analysis, when determining a diagnosis of classical Hodgkin lymphoma as compared with AIDS‐related NHLs and other aggressive B‐cell NHLs.

FC is a cornerstone technology in the diagnosis and follow‐up of lymphoproliferative disorders, including follicular lymphoma (FL) and primary mediastinal large B‐cell lymphoma (PMBCL). Its utility stems from the ability to rapidly and quantitatively assess multiple antigenic markers on individual cells, enabling precise immunophenotypiccharacterisation, detection of clonal populations, and assessment of disease burden in a variety of specimen types, including peripheral blood, bone marrow, and tissue aspirates [58, 59, 62, 67]. In the diagnosis of FL, FC is used to identify a clonal B‐cell population with a characteristic immunophenotype: CD19+, CD20+, CD10+, BCL2+, and surface immunoglobulin light chain restriction. The detection of CD10 and BCL2 co‐expression is particularly helpful in distinguishing FL from other indolent B‐cell lymphomas, although some cases may lack one or both markers, necessitating integration with morphologic and molecular data [62]. FC is especially valuable in small‐volume samples, such as fine needle aspirates or core biopsies, where it can provide rapid and sensitive detection of neoplastic cells, often outperforming cytology alone in sensitivity and specificity [28, 67, 81]. In bone marrow staging, FC can detect minimal involvement that may be missed by histology, aiding in accurate disease staging and risk stratification.

For primary mediastinal large B‐cell lymphoma (PMBCL), FC faces unique challenges. PMBCL cells are often sparse in aspirates and may be masked by a prominent reactive background. However, advances in multiparametric panels and algorithmic approaches have improved the ability to distinguish PMBCL from other “Hodgkin‐like” entities. PMBCL typically shows a B‐cell phenotype (CD19+, CD20+, CD79a+) with variable CD30 expression and absence of CD10, and may lack surface immunoglobulin. The analysis of background T‐cell and B‐cell populations can further refine the differential diagnosis, especially when combined with morphologic and molecular data [48]. While FC is less sensitive for direct tumour cell detection in PMBCL compared to FL, it remains a useful adjunct for immunophenotypic profiling and exclusion of other lymphoproliferative disorders.

Across lymphoproliferative disorders, FC enables the identification of abnormal lymphoid populations by their antigenic expression patterns, facilitating lineage assignment (B‐cell, T‐cell, NK‐cell), detection of aberrant phenotypes, and assessment of clonality. It is indispensable for subclassification of non‐Hodgkin lymphomas, including mantle cell lymphoma, marginal zone lymphoma, and chronic lymphocyticleukaemia, each with distinct immunophenotypic profiles. FC also supports the diagnosis of T‐cell and NK‐cell neoplasms, where clonality can be inferred from aberrant antigen loss or restricted T‐cell receptor expression. In follow‐up and MRD assessment, FC offers high sensitivity for detecting low‐level disease, particularly in peripheral blood and bone marrow. MRD monitoring is increasingly important in guiding therapy and predicting relapses, especially in aggressive lymphomas and in the context of novel immunotherapies. FC can also assess prognostic markers (e.g., CD38, CD56, ploidy status), therapeutic targets (e.g., CD20 for anti‐CD20 therapy), and multidrug resistance markers, providing actionable information for personalised treatment strategies [68].

The integration of FC with morphology, immunohistochemistry, and molecular studies is essential for comprehensive lymphoma diagnosis, especially as clinical practice shifts toward less invasive sampling methods. The hematopathology community, as discussed in the medical literature, supports the use of FC as a key component of the diagnostic workup for lymphoproliferative disorders, particularly when tissue volume is limited [67, 81]. While surgical biopsies remain the gold standard for full characterisation, FC enables accurate diagnosis and subclassification in many cases, reducing the need for invasive procedures. Thus, FC is a high‐precision tool for the diagnosis, classification, staging, and follow‐up of FL, PMBCL, and other lymphoproliferative disorders. Its strengths include rapid multiparametric analysis, high sensitivity for clonal populations, and utility in small‐volume samples. Limitations include reduced sensitivity in certain entities (e.g., PMBCL) and the need for integration with other diagnostic modalities. Ongoing advances in antibody panels, data analysis algorithms, and integration with molecular techniques continue to expand the role of FC in hematopathology.

Disparities in flow cytometry availability are significant between high‐income countries and low‐/middle‐income countries, where the healthcare systems are lacking access to advanced diagnostic modalities such as multiparametric flow cytometry due to economic resource constraints, limited infrastructure and insufficient trained personnel. This impacts the ability to apply diagnostic algorithms and ancillary techniques that are standard in high‐income countries. Despite these limitations, our review on debating the use of flow cytometry for diagnosis of HIV‐associated HLs remains applicable in settings where flow cytometry is not available by offering insights into the immunophenotypic features of HL and HIV associated HL. Such a review clarifies the essential markers and the morphologic context required for diagnosis, which can guide the pathologists for low‐ and middle‐income countries to optimise the use of available resources such as histopathology and immunohistochemistry, which remain the gold standard for HL diagnosis. Moreover, understanding the flow cytometry approach can help in building up future capacities, to better allocate resources, and can be fundamental for improving the diagnostic infrastructure based on other countries’ experiences, which are summarised in such studies [32, 67].

## 4 Conclusion

In summary, the immunophenotype of HRS cells by FC does not fundamentally change in HIV‐related classical Hodgkin lymphoma, but the background immune cell composition is significantly altered, with fewer CD4+ T cells, more CD8+ T cells, and increased macrophages. These microenvironmental changes are the most characteristic features distinguishing HIV‐related from non–HIV‐related classical Hodgkin lymphoma, in FC analysis.

cHL in HIV‐positive patients frequently exhibits a higher proportion of EBV‐positive Reed–Sternberg cells, overexpression of EBV and a tumour microenvironment characterised by a significant reduction in CD4+ T cells, increased CD8+ T cells, and an M2 macrophage predominance. This results in a CD4:CD8 ratio that is reversed or very low, which is not the typical T‐cell‐rich, CD4‐dominant background in immunocompetent patients with cHL. Moreover, HIV‐associated cHL neoplastic cells may express CD20 with more frequency and have more numerous neoplastic cells in comparison to HIV‐negative cases, while the hallmark immunophenotype (CD30+, CD15+, B‐cell markers weak/absent, CD45–) is usually preserved.

In patient sample analysis, multiparametric FC assays for cHL have demonstrated sensitivity ranging from 85% to 95% and specificity of 98.2% in lymph node and biopsy specimens [69]. The American Society for Clinical Pathology and the College of American Pathologists note that FC is not routinely used for cHL diagnosis, but whenoptimised, it can achieve high sensitivity and specificity [69].

Several studies emphasise the importance of FC detection of cHL rapidly and effectively, adding diagnostic value to these small biopsies. FC is an important tool for clinical decision‐making in the management of HL patients, providing a noninvasive and accurate biomarker evaluation [32, 65, 74]. For these considerations, we can conclude that FC is a very useful research tool, and the clinical cases presented in this paper indicate its importance in the rapid diagnosis of cHL as well.

## Author Contributions

**Tendani Gaolathe:** investigation, validation. **Andrew K. Ndlovu:** investigation, visualization. **Diana Cenariu:** validation, investigation. **Adrian‐Bogdan Tigu:** investigation, validation. **Maria Santa:** investigation, validation, methodology. **Diana Gulei:** methodology. **Patricia Rantshabeng:** investigation, conceptualization, formal analysis. **Khalid Abdelrahman:** investigation, validation. **Ioana Rus:** methodology. **Marc Damian:** investigation, conceptualization. **Madalina Nistor:** methodology. **Cristina Selicean:** methodology. **Bogdan Fetica:** formal analysis, validation. **Sanda Buruiana:** methodology, investigation, writing – original draft. **Horia Bumbea:** methodology, formal analysis. **David Kegyes:** methodology. **Victor Tomacinschii:** validation, methodology. **Mihnea Zdrenghea:** formal analysis, software, supervision. **Anamaria Bancos:** methodology. **Rand Bilal:** conceptualization, methodology. **Cristian Jinca:** methodology. **Cristina Stefan:** validation, writing – review and editing. **Ciprian Tomuleasa:** supervision, formal analysis, visualization, writing – review and editing, data curation. **Jonathan Fromm:** resources, supervision, formal analysis, software.

## Funding

This work was supported by the Romanian Government, PNRR/2022/C9/MCID/18; 760278/26.03.2024, PN‐IV‐P8‐8.3‐ROMD‐2023‐0036; the European Commission, Proposal Number 101227725 – “Advancing in the CHallenge of Universitatea de Medicină şi Farmacie Iuliu Haţieganu Cluj‐Napoca,” No 32154/9/16.12.2024, PCD – Maria Santa.

## Ethics Statement

The authors have nothing to report.

## Consent

All authors have read and agreed to the publication. No patient information is reported, and thus no patient consent is applicable.

## Conflicts of Interest

The authors declare no conflicts of interest.

## Supporting information

Supporting Information: 1.

Supporting Information: 2.

Supporting Information: 3.

## Data Availability

The data that support the findings of this study are available on request from the corresponding author. The data are not publicly available due to privacy or ethical restrictions.
