# Supplementary material for: Integrating Flow Cytometry in the Diagnostic Work of HIV‐Associated Hodgkin's Lymphomas
Source: J Cell Mol Med. 2026 May 5;30(9):e71143. doi: 10.1111/jcmm.71143 (PMC13143871; doi:10.1111/jcmm.71143)
Supplement: Supplementary file 1 — Supporting Information: 1. [file JCMM-30-e71143-s003.docx]

As a proof-of-concept case, we report a 36-year-old male with a 10-year history of HIV infection (on antiretroviral therapy, current CD4 count 250 cells/µL) presents with persistent cervical lymphadenopathy, night sweats, and unintentional weight loss over two months. In the initial evaluation, the physical exam showed firm, non-tender lymph nodes in the left cervical chain (2–3 cm). Laboratory findings revealed mild anemia (Hb 10.8 g/dL), elevated ESR (90 mm/hr), and LDH mildly elevated. The initial PET-CT reveals hypermetabolic activity in cervical, mediastinal, and para-aortic lymph nodes. At lymph node biopsy, histopathology: Effacement of nodal architecture with scattered large Reed–Sternberg cells in a background of small lymphocytes, eosinophils, and histiocytes. Immunohistochemistry revealed Reed–Sternberg cells positive for CD15, CD30, and PAX5 (weak); negative for CD45, consistent with a diagnosis of cHL (mixed cellularity subtype). HIV status was at diagnosis under control on anti-retroviral therapy (ART), viral load undetectable. Although Hodgkin lymphoma is typically diagnosed histologically, flow cytometry plays a key role in monitoring immune status and treatment effects in HIV-positive patients. The bone marrow and peripheral blood FC (if marrow involvement suspected) is used to valuate for abnormal B-cell populations or immunodeficiency-related dysregulation.

For monitoring timeline, at baseline (before therapy): Flow cytometry shows CD4 count of 250 cells/µL and CD8 count of 800 cells/µL (CD4/CD8 ratio = 0.31). No circulating abnormal B-cell population. During chemotherapy (ABVD regimen): Flow cytometry performed every 2–3 cycles to monitor CD4 recovery and lymphocyte subset shifts. After 3 cycles: CD4 drops to 180 cells/µL (expected chemotherapy effect). No evidence of abnormal B-cell clones. Post-treatment (6 months later), CD4 count improves to 320 cells/µL with ART continuation. Flow cytometry remains negative for abnormal B-cell populations. PET-CT confirms complete metabolic remission.

This case report presents a clinical scenario involving an HIV-positive patient diagnosed

with classical Hodgkin lymphoma (cHL), with emphasis on the role, strengths, and limitations of flow cytometry (FC) in clinical decision-making.

1. Diagnostic Context

The patient, a 36-year-old male with long-standing HIV infection on effective antiretroviral therapy, presented with systemic symptoms and generalized lymphadenopathy. Definitive diagnosis of mixed cellularity classical Hodgkin lymphoma was established through lymph node biopsy and immunohistochemistry demonstrating Reed–Sternberg cells positive for CD15 and CD30, with weak PAX5 expression and CD45 negativity.

Flow cytometry is not a primary diagnostic modality for classical Hodgkin lymphoma, as Reed–Sternberg cells are scarce and often not detectable in suspension-based assays. Therefore, histopathology remains the gold standard for diagnosis.

1. Baseline Utility of Flow Cytometry.

At baseline, flow cytometric analysis demonstrated a CD4 count of 250 cells/µL, a CD8 count of 800 cells/µL, and a CD4/CD8 ratio of 0.31, with no abnormal circulating B-cell populations identified.

Clinical significance:

- Establishes baseline immune competence prior to chemotherapy.

- Helps exclude concurrent HIV-associated non-Hodgkin lymphomas.

- Assists in infection risk stratification and treatment planning.

3. Role During Chemotherapy

Serial flow cytometry during ABVD chemotherapy showed a decline in CD4 count to 180 cells/µL, consistent with treatment-related immunosuppression, without evidence of clonal B-cell populations.

Clinical significance:

- Differentiates expected chemotherapy effects from pathological immune deterioration.

- Guides opportunistic infection prophylaxis.

- Supports continuation of combined ART and chemotherapy.

4. Post-Treatment Monitoring

Six months after completion of therapy, flow cytometry showed CD4 recovery to 320 cells/µL with persistent absence of abnormal B-cell populations. This correlated with PET- CT findings of complete metabolic remission.

Clinical significance:

- Demonstrates immune reconstitution.

- Supports sustained oncologic remission.

- Confirms effective HIV control in the post-treatment phase.

5. Overall Re-assessment

This case highlights that flow cytometry in classical Hodgkin lymphoma is best utilized as an adjunct tool for immune profiling and longitudinal monitoring, particularly in immunocompromised patients. While it does not replace histologic diagnosis, it provides essential insights into immune status, treatment tolerance, and recovery.

Conclusion

Flow cytometry plays a complementary yet crucial role in the management of Hodgkin lymphoma in HIV-positive patients by informing immune assessment, monitoring therapy- related effects, and supporting long-term follow-up.
