# Supplementary material for: Integrating Flow Cytometry in the Diagnostic Work of HIV‐Associated Hodgkin's Lymphomas
Source: J Cell Mol Med. 2026 May 5;30(9):e71143. doi: 10.1111/jcmm.71143 (PMC13143871; doi:10.1111/jcmm.71143)
Supplement: Supplementary file 2 — Supporting Information: 2. [file JCMM-30-e71143-s001.docx]

A 51-year-old male with a 28-year history of HIV infection (on antiretroviral therapy, current CD4 count 300 cells/µL) presents with rapidly enlarging cervical and axillary lymph nodes, intermittent fever, and weight loss over three weeks. At initial evaluation, he presented with multiple firm, non-tender lymph nodes in the cervical and axillary regions (up to 4 cm in diameter). Laboratory findings showed elevated LDH, mild anemia (Hb 11.0 g/dL), and elevated β2- microglobulin. PET-CT shows extensive hypermetabolic lymphadenopathy involving cervical, mediastinal, and abdominal regions. Excisional lymph node biopsy revealed diffuse effacement of nodal architecture by sheets of large atypical lymphoid cells. Immunohistochemistry demonstrated tumor cells positive for CD19, CD20, CD79a, BCL-6, and MUM1 and negative for CD3 and CD10, consistent with diffuse large B-cell lymphoma (DLBCL), activated B-cell subtype.

Flow cytometry was used both diagnostically and for ongoing monitoring of minimal residual disease (MRD) in DLBCL. Flow cytometry was used as an adjunct diagnostic tool for immunophenotypic characterization and for immune monitoring related to HIV infection and chemotherapy-associated immunosuppression. Flow cytometry was not used as a standalone tool for MRD assessment or for guiding treatment decisions. Baseline flow cytometry identified a monoclonal B-cell population expressing CD19, CD20, and kappa light chain restriction, supporting the histopathologic diagnosis. Serial flow cytometry during treatment demonstrated no detectable circulating clonal B-cell population; however, treatment response and remission status were assessed according to PET-CT imaging and clinical criteria.

FC panels used for diagnostic immunophenotyping as markers CD19, CD20, CD22, CD79a, CD10, CD5, CD45, kappa/lambda light chains (Figure 4). The purpose was to identify clonal B-cell populations with restricted light chain expression, confirming the diagnosis of B-cell lymphoma. Immune reconstitution monitoring is used as markers CD3, CD4, CD8, CD19, CD16/CD56. The purpose is to assess CD4/CD8 ratio and monitor lymphocyte subset recovery during R-CHOP chemotherapy and ART. The MRD markers (CD19, CD20, CD10, CD45, kappa/lambda, and BCL-2) detect residual clonal B-cell populations in peripheral blood or bone marrow post-therapy. The baseline (before therapy) FC shows monoclonal B-cell population expressing CD19+, CD20+, and kappa light chain restriction. CD4 count is 300 cells/µL, CD8 count is 950 cells/µL (CD4/CD8 ratio = 0.31). During chemotherapy (R-CHOP regimen), FC was performed after every 2 cycles; and after 3 cycles, no monoclonal B-cell population detected, but CD4 count drops to 180 cells/µL (chemotherapy-related immunosuppression). Post-treatment (6 months later) FC was negative for clonal B-cell population. CD4 count improves to 340 cells/µL, CD8 to 900 cells/µL (CD4/CD8 ratio = 0.38). PET-CT confirms complete remission.

Diffuse large B-cell lymphoma is a biologically heterogeneous disease, and current

International guidelines do not recommend flow cytometry-based minimal residual disease (MRD) assessment as standard clinical practice. Unlike indolent lymphomas or acute leukemias, DLBCL typically lacks a consistent circulating tumor population or stable immunophenotypic marker suitable for reliable MRD detection. Current response assessment in DLBCL relies primarily on functional imaging, particularly PET-CT, using the Lugano classification. Both NCCN and ESMO guidelines emphasize that PET-CT findings, in conjunction with clinical evaluation, should guide therapeutic decisions and define remission status.

Flow cytometry may detect circulating clonal B cells in select cases; however, absence of

detectable disease by flow cytometry does not equate to molecular remission, nor does detection of rare clonal populations necessarily indicate active disease. Overreliance on flow cytometry for MRD assessment in DLBCL may therefore lead to misinterpretation and inappropriate clinical decision-making. Emerging techniques such as next-generation sequencing-based circulating tumor DNA (ctDNA) analysis demonstrate promise for MRD detection in DLBCL, but these approaches remain investigational and are not yet incorporated into routine clinical practice.

In HIV-associated DLBCL, flow cytometry retains an important role in immune monitoring, particularly for assessment of CD4/CD8 counts and infection risk during immunochemotherapy. In this context, flow cytometry provides supportive information but should be interpreted separately from lymphoma response assessment. This case highlights the importance of aligning diagnostic and monitoring strategies with established guidelines. Flow cytometry should be regarded as complementary to histopathology and imaging rather than a determinant of MRD or remission status in DLBCL.
