# Supplementary material for: Integrating Flow Cytometry in the Diagnostic Work of HIV‐Associated Hodgkin's Lymphomas
Source: J Cell Mol Med. 2026 May 5;30(9):e71143. doi: 10.1111/jcmm.71143 (PMC13143871; doi:10.1111/jcmm.71143)
Supplement: Supplementary file 3 — Supporting Information: 3. [file JCMM-30-e71143-s002.docx]

A 31-year-old man with a known HIV infection (diagnosed 6 years ago) presents with rapidly enlarging cervical lymph nodes, night sweats, and weight loss over 3 weeks. Medical history included previous therapy with ART, but recently nonadherent. Most recent CD4 count (2 months ago): 180 cells/µL and the viral load showed 150,000 copies/mL. The physical examination revealed multiple firm, non-tender lymph nodes in the neck and axillae and enlarged spleen and liver. In the diagnostic work-up, the lymph node biopsy shows sheets of medium-sized lymphoid cells with high mitotic activity and numerous apoptotic bodies (“starry-sky” pattern). Flow cytometric immunophenotyping of the lymph node suspension (Figure 5) reveals clonal B-cell population expressing positive staining for CD19, CD20, CD10, CD79a, surface IgM, BCL6; with light-chain restriction (κ or λ); as well as negative staining for CD5, CD23, TdT. The immunophenotype was consistent with Burkitt lymphoma (BL) - a high-grade B-cell non-Hodgkin lymphoma often associated with HIV. Cytogenetic confirmation (FISH) showed t(8;14)(q24;q32), with a MYC translocation. Burkitt lymphoma commonly occurs in patients with moderate immunosuppression (CD4 counts &gt;100 cells/µL), consistent with this case. FC was used in the follow-up and after initiation of intensive chemotherapy (CODOX-M/IVAC) and optimization of ART, for monitoring the immune system (HIV follow-up), peripheral blood FC quantifies CD4⁺ and CD8⁺ T-lymphocyte subsets to assess immune recovery and guide ART. As an example, CD4 count rising from 180 → 450 cells/µL after 6 months suggests immune reconstitution. For MRD monitoring (lymphoma follow-up), FC on bone marrow or peripheral blood detects residual clonal B-cell populations with the same immunophenotype as the initial tumor (CD10+, surface IgM+, light-chain restriction). A negative MRD result indicates molecular remission. In the clinical outcomes, after 6 cycles of chemotherapy and continued ART, FC shows no residual clonal B-cells in marrow, CD4 count improves to 420 cells/µL and the patient remains in complete remission at 12 months.
